# Modelling concrete and abstract concepts using brain-constrained deep neural networks

**DOI:** 10.1007/s00426-021-01591-6

**Published:** 2021-11-11

**Authors:** Malte R. Henningsen-Schomers, Friedemann Pulvermüller

**Affiliations:** 1 Department of Philosophy of Humanities, Brain Language Laboratory, Freie Universität Berlin, Habelschwerdter Allee 45, 14195 Berlin, Germany; 2 Berlin School of Mind and Brain, Humboldt-Universität zu Berlin, Berlin, Germany; 3 Einstein Center for Neurosciences, Berlin, Germany; 4 Cluster of Excellence ‘Matters of Activity. Image Space Material’, Humboldt-Universität zu Berlin, Berlin, Germany

## Abstract

A neurobiologically constrained deep neural network mimicking cortical area function relevant for sensorimotor, linguistic and conceptual processing was used to investigate the putative biological mechanisms underlying conceptual category formation and semantic feature extraction. Networks were trained to learn neural patterns representing specific objects and actions relevant to semantically ‘ground’ concrete and abstract concepts. Grounding sets consisted of three grounding patterns with neurons representing specific perceptual or action-related features; neurons were either unique to one pattern or shared between patterns of the same set. Concrete categories were modelled as pattern triplets overlapping in their ‘shared neurons’, thus implementing semantic feature sharing of all instances of a category. In contrast, abstract concepts had partially shared feature neurons common to only pairs of category instances, thus, exhibiting family resemblance, but lacking full feature overlap. Stimulation with concrete and abstract conceptual patterns and biologically realistic unsupervised learning caused formation of strongly connected cell assemblies (CAs) specific to individual grounding patterns, whose neurons were spread out across all areas of the deep network. After learning, the shared neurons of the instances of concrete concepts were more prominent in central areas when compared with peripheral sensorimotor ones, whereas for abstract concepts the converse pattern of results was observed, with central areas exhibiting relatively fewer neurons shared between pairs of category members. We interpret these results in light of the current knowledge about the relative difficulty children show when learning abstract words. Implications for future neurocomputational modelling experiments as well as neurobiological theories of semantic representation are discussed.

## Introduction

Here, we address the question how concepts are represented in the mind^1^ and brain. We do this by specifying putative neurobiological correlates of concepts, spelt out in the language of the brain, that is, in terms of nerve cells, neuronal groups and their structure and connectivity. We specifically focus on the mechanisms by which specific instances of perceptions and actions can lead to the build-up of conceptual category representations which do not stand for the individual entities, i.e. perceptuo-motor experiences or memories thereof, but, instead, for whole classes of objects or actions. We also address putative differences in the neurobiological mechanisms underlying concrete and abstract concepts.

This investigation is performed by mimicking the learning of actions and perceptions within a neuronal network model, which replicates structural and functional aspects of relevant anatomical structures of the human brain. We stimulate this brain-constrained network model (Pulvermüller et al., 2021) with stimulation patterns of different degrees of similarity and observe and describe the resulting assemblage of neuronal circuits within the network. We then draw careful conclusions on the putative mechanistic basis of concepts and putative differences between abstract and concrete concepts, in terms of their underlying neuronal circuits.

Current semantic theories do already offer multiple ways to address conceptual mechanisms at abstract levels. Semantic feature models use pairs of semantic features and feature values to characterize concepts. A BACHELOR (we use terms in capitals to refer to conceptual entities) would thereby be characterized as + HUMAN, + MALE, + ADULT and -MARRIED. Features can be concrete or abstract, so that a concrete concept, such as REDNESS, would exhibit the feature + RED and abstract concepts, such as CAUSE or DEMOCRACY, are characterized by the abstract features + CAUSAL or + DEMOCRATIC. This sometimes circular approach delivers systematic descriptions of meaning and may allow for economic descriptions of the semantics of huge vocabularies with a limited set of features. However, it does not address the question of how concepts relate to the real world in which children have to learn at least some key concepts from experiences (Harnad, 1990; Vincent‐Lamarre et al., 2016). And even if one is inclined to hold that concepts are given to humans a priori, there would be need to connect concrete objects or actions with the internal a priori entities by learning. It has been pointed out that the semantic feature approach does not offer an explanation for such conceptual learning and, apart from this issue, is at variance with a range of facts known from language use (Lakoff, 1987; Löbner, 2013).

As an alternative model of conceptual relationships and content, distributional semantic models use information about the frequent contexts in which words expressing concepts appear for defining these concepts (Landauer & Dumais, 1997; Lund & Kevin, 1997; Lund & Burgess, 1996; see Lenci, 2018, for a recent review). This strategy rests on the assumption that conceptual and semantic knowledge are due to the memorized contexts in which words appear (see Schwanenflugel et al., 1988). However, in order to extract meaning from context, it is necessary to have semantic and conceptual information for the contexts available in the first place and this is not explained by an account defining concepts in terms of contexts per se (Searle, 1980). Therefore, distributional information alone cannot suffice to explain concepts, as it runs into the so-called symbol grounding problem (Harnad, 1990). The same argument also applies for semantic feature models, where each semantic feature would need to be grounded in the entities it is about.

Note that, whereas semantic theories defining the meaning of symbols in terms of symbolic context or other symbols (e.g. for semantic features) run into the grounding problem, semantic theories relying primarily on grounding are themselves problematic, because most words and symbols (ca. 80%) are typically learnt not in real life situations where reference objects and actions are present in the environment of the communicating individuals, but rather from texts (Kintsch, 1974, 1998). So-called ‘hybrid models’ of semantics and concepts (Andrews et al., 2009; Davis & Yee, 2021; Glenberg & Robertson, 2000; Harnad, 1990; Louwerse & Jeuniaux, 2010) take into account both relevant facts, that at least some concepts and symbols require conceptual ‘grounding’ in specific sensorimotor information from the world, that is, in concept-related objects, actions or their features, and that, after such grounding has happened, distributional or other types of learning relating symbols to symbols can function via contextual transfer of conceptual information. The learning of symbolic meaning by way of previously grounded symbols is sometimes called ‘indirect grounding’, ‘grounding transfer’ or ‘symbolic theft’ and is now supported by ample evidence both from behavioural experiments and computational models (e.g. Cangelosi & Riga, 2006; Cangelosi et al., 2002; Günther, et al., 2020a, b) and even cognitive robotics (Cangelosi & Stramandinoli, 2018). According to one estimate, a minimum of 10% of words of a vocabulary must be directly grounded in entities in the world, so as to allow for conceptual learning based on distributional learning and ‘symbolic theft’ (Blondin Massé et al., 2008). Therefore, the question remains how at least a basic ‘grounding kernel’ of directly grounded concepts can be established.

**Fig. 1:**
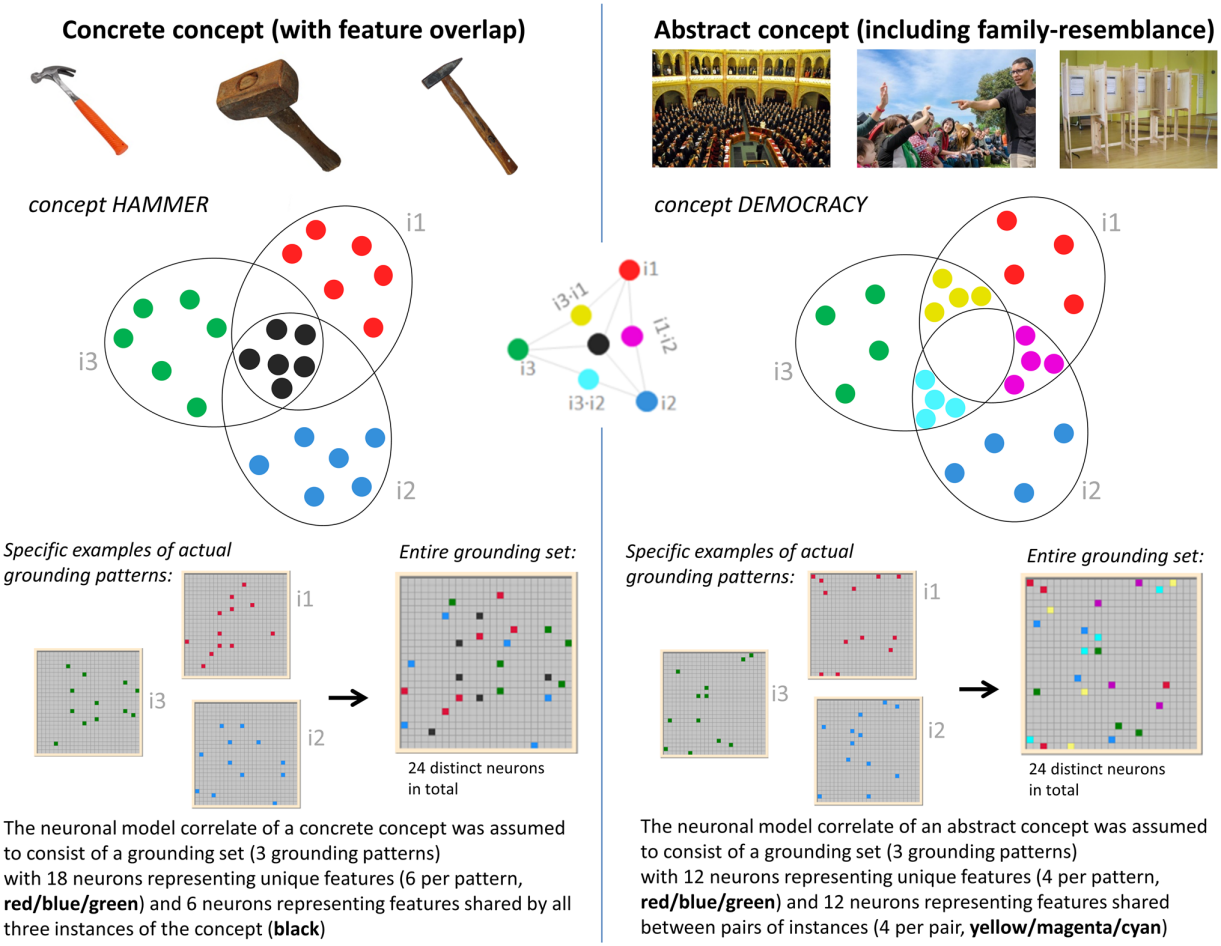
Schematic illustration of a structural difference between concrete (left) and abstract (right) concepts (semantic feature overlap vs. family resemblance). We model the semantic features of any given concept as being represented in 3 grounding patterns per modality (sensory and motor), with 12 neurons per grounding pattern (i.e. 24 per grounding pattern across both modalities). Note, however, that we only show one modality here for simplification. However, the procedures were identical for grounding patterns used as input to *V1 and *M1_L_ (see also Fig. 2D). Left panel: concrete concepts were modelled as containing 12 neurons per grounding pattern in total, 6 shared between all three (representing semantic features) and 6 unique to each instance (representing instance-specific perceptual or action-related features). In the example of HAMMER, the 6 shared and therefore ‘semantic’ neurons represent general visual features, such as shape features including long handle, head attached at a 90-degree angle along with general action-related ones, including typical motor trajectories characterizing the beating with a hammer. The 6 instance-specific sensory and motor neurons represent unique features of each hammer exemplar, including idiosyncratic properties (e.g. differing sizes, materials, shapes of the head, presence or absence of a wedge), along with specificities of the way each hammer requires sensorimotor adjustment to these individual properties when being used. Right panel: abstract concepts were modelled by an implementation of family resemblance, whereby each grounding pattern of an instance is represented by 12 neurons, 4 shared between two instances and 4 unique to only one instance. In the example of DEMOCRACY, pairwise shared neurons might represent hand actions involved in casting a vote (shared between i2/i3) or the visual image of several people coming together (shared between i1/i2). Unique features might represent differences in the hand movements for raising ones hand vs. throwing a ballot in a ballot box (i2 vs i3) or differences in the size and layout between an official parliament room and a smaller room where people cast votes in an informal setting. For each panel, the top half of the figure with overlapping ovals shows a schematic depiction of how we modelled differences between concrete and abstract concepts, whereas the lower half shows specific examples of actual grounding patterns (12 neurons active out of a 25 × 25 grid) used in the model; see “Methods” for details. Photographs were obtained from the world wide web, and were published under a CC0 license (https://creativecommons.org/share-your-work/public-domain/cc0/)

The concept DEMOCRACY, for example, can be explained purely through verbal description by making reference to the concepts of PARLIAMENT, VOTING, EQUALITY, BALLOTS, BALLOT BOXES, etc. However, these in turn would require grounding on specific sensorimotor information again—the information that democracy involves voting and that votes can be recorded on a ballot is of no help if one does not know what voting and ballots are. Alternatively, DEMOCRACY could be directly grounded through sensory experiences, e.g. images of parliament meetings, rooms of people informally voting by raising their hands, parliament buildings and/or motor experiences, such as knowing what it is like to cast a ballot, raising one’s hand or similar (see also Fig. 1 for an illustration).

For concrete concepts, conceptual grounding is straightforward to explain. The concept of a specific person can be grounded in the visual image of that person or in specific features of her or him, such as a particular shape of the mouth, timbre of the voice or odor. Similarly, a categorial concept can be grounded in typical and therefore frequently encountered features of the category members, for example the fur, long tail and pointed ears of instances of the concept CAT. It has been claimed that categories are typically characterized by shared features of all category members (Locke, 1847) and, although this position has been criticized repeatedly (Lakoff, 1987; Rosch & Mervis, 1975; Wittgenstein, 1953), it provided a useful rationale for the semantic feature approach mentioned above. In some semantic frameworks, it is established to describe the meaning of category terms by way of shared semantic features (Löbner, 2013), even though this strategy seems to work reasonably well only for quite concrete and narrowly defined concepts (see “Discussion” below).

Large categorial concepts (such as ANIMAL, GAME) and abstract words more generally can, in many cases, not be easily described in terms of common sensory or motor and, hence, semantic, features (Yee, 2019). It has been argued that peculiarities of abstract concepts relate to their different ontological status that “abstract entities are not in spacetime whereas concrete entities are” (Dummett, 1981; Hale, 1988). However, leaving aside the highly philosophical question about their ontological status, it is undeniable that both abstract and concrete concepts are in fact concepts and therefore, in one sense, not in the world, where space and time apply, but rather ‘in the mind’. In addition, both concrete and abstract concepts need to be applied in real life to make claims or confirm vs. reject them. After all, whether the statement “this is DEMOCRACY” (or “DEMOCRATIC”) is correctly applied in light of the practice of voting at a specific election is an empirical issue—and this question is comparable to (although more complex than) that of whether this animal is a CAT. Hence, as statements with both abstract and concrete terms need to potentially undergo verification or falsification, there need to be criteria for matching concepts with entities in the world or their features (Frege, 1892; Locke, 1847). Psychological experiments where subjects are asked to list their situational associations for concrete and abstract concepts further confirm that both are intrinsically linked to background situational information and that these links are central to their content (Barsalou & Wiemer-Hastings, 2005). Therefore, it is established that also abstract terms need to be grounded, although their grounding process may somewhat differ from that of concrete terms.

In search of specific differences between concrete and abstract concepts and their grounding in ‘world relationship’, psychologists and linguists have highlighted several features. The dual coding theory postulates that abstract concepts and words are represented in a verbal system, whereas only concrete ones are represented by both verbal and imagistic codes (Paivio, 1971, 1991). However, given the situational links of abstract concepts documented empirically (Barsalou & Wiemer-Hastings, 2005), it appears partly problematic to exclude an imagistic code for abstract entities. A difference may lie in qualitatively different imagistic codes for the two concept types, with concrete concepts offering relatively more sensory and motor associations and abstract terms more emotional–affective information associated with them (Kousta et al., 2011; Vigliocco et al., 2014). However, this position seems to be driven by concepts that are abstract because they relate to internal emotional states, for example JOY, SORROW, LOVE and AGONY, but not abstract mental terms, such as LOGIC, CAUSE, NUMEROCITY and PROOF. A similar perspective views external and internal attributes as relatively more crucial for concrete and abstract concepts, respectively, based on the fact that study participants tend to describe concrete concepts (e.g. BIRD) using concrete action- and perception-related words (“beach”, “fly”, “food”), whereas, for abstract concept description (e.g. TRUE), more abstract (“introspective”) terms are applied (“belief”, “think”, “idea”) (Barsalou & Wiemer-Hastings, 2005). However, this proposal rests on the presupposition that introspection offers a pathway to semantic grounding of novel unknown symbols, a claim that is controversial (Baker & Hacker, 2008; Gebauer, 2013). In one view, the grounding of inner states and emotions relies on neurocognitive systems for motor movements and actions (Dreyer & Pulvermüller, 2018; Moseley et al., 2012), thus, casting doubt on the feasibility of inner vs. external grounding distinctions.

All of these aforementioned approaches interlink different domains of semantic content (linguistic vs. imagistic, sensorimotor vs. emotional, external vs. internal) with concrete and abstract concepts, but do not postulate a principal structural difference between them. From a grounding perspective, one may argue that, possibly, primarily concrete concepts are grounded directly in action and perception, whereas grounding of abstract concepts is indirect, through context, a position that seemingly fits with experimental results (Günther, et al., 2020a, 2020b; Wiemer‐Hastings & Xu, 2005; Zdrazilova & Pexman, 2013; Zwaan, 2016). Still, also this position may not capture the most important differences, especially as very concrete terms are easily derived from contextual information (e.g. Harnad’s famous example ZEBRA, grounded in the conjunction of STRIPED and HORSELIKENESS) and, clearly, both abstract and concrete concepts are amenable to an analysis in terms of distributional semantics. That abstract terms are exclusively grounded indirectly in contexts may not appear as a fully convincing proposal, because in order to ground an abstract term like “truth” indirectly in contexts including “belief”, “think” and “idea”, at least some of the equally abstract context words need to be grounded directly. In order for this approach to work, it would be necessary to assume that at least some abstract concepts are grounded in the context of expressions related to objects and actions (Stramandinoli et al., 2017), but this position raises the question why, in this case, grounding could not be direct, that is, in the object perceptions and action performances themselves. Such direct grounding of highly abstract concepts is certainly possible, as recently illustrated using the example concept of CAUSATION (Pulvermüller, 2018b) and regression to the mean. These arguments render the idea of differential direct vs indirect grounding of concrete and abstract concepts not fully convincing.

A structural description of the difference between concrete and abstract concepts goes back to the observation of a property called family resemblance (Baker & Hacker, 2008; Wittgenstein, 1953). As mentioned above, the classic approach to category structure, that a distinctive set of semantic features are shared between the members of a category, fails in case of large or relatively abstract categories. For example, consider the concept BIRD, where some category members indeed lack the core features of FLYING and HAVING FEATHERS; or the concept GAME, where features, such as GROUP ACTIVITY, PLEASANTNESS and COMPETITIVITY apply to subgroups of instantiations, but not to the entire set of activities falling under the term. There is a tension amongst semantic frameworks, where one fraction advocates, in spite of these counterexamples, the classic idea of common semantic features defining a concept, while the other fraction advocates the general applicability of family resemblance. Pulvermüller (2013, 2018b) proposed to apply the family resemblance feature for distinguishing abstract from concrete concepts and for characterizing a gradual abstract–concrete dimension. Concrete concepts are seen as sharing a set of common semantic features, whereas abstract ones are characterized by partial feature sharing, so that semantic features are common to just a subset of instantiations falling under a given category.

Figure 1 schematically illustrates this difference between full vs. partial semantic feature overlap. In this display, each small circle represents an individual neuronal element thought to carry one specific perceptual/sensory or action-related/motor feature activated by one or more instances of a concept. One can classify these neurons into unique neurons (present in only one instance of the category) and shared neurons (present in more than 1 instance). The latter will also be called ‘semantic feature’ neurons here. Concrete concepts or categories (we use these terms interchangeably), are characterized by a core set of semantic neurons shared by all (or almost all) instances, whereas abstract concepts include no (or a minimal) core set, but rather semantic feature neurons only partially shared by a subset of instances (in this case, 2 out of 3). We take this structural difference as a key for the distinction between concrete and abstract categories from which other differentiating features (such as the tightness or looseness of the semantic links to real world instances) may follow.

We would like to remark that, when contrasting the structure of abstract and concrete concepts using simple paradigmatic examples, we see these extremes as ends of a continuum, not as a binary distinction. There is broad agreement that—generally speaking—concrete concepts tend to be characterized by many shared features and hence are quite homogenous in their feature distribution, whereas abstract concepts are more heterogeneous. Several other authors have made theoretical distinctions that seem to rely on this important aspect. For example, Lupyan and Mirman (2013) conducted a study with aphasia patients and distinguished low-dimensional vs. high-dimensional categorization tasks. In their study, “high-dimensional” categories had many shared semantic features among category members, whereas “low-dimensional” categories shared only one or a few features. A similar distinction has also been made by other authors (Kloos & Sloutsky, 2008; Sloutsky, 2010), calling it statistical density. Note that both these proposals from other authors bear some resemblance to the distinction between semantic feature overlap and family resemblance made here; a crucial difference still remains, however, as family resemblance entails a qualitatively different semantic structure and sharing of semantic features. Aside from this point, a rating study by Granito et al. (2015) showed that while quantitative differences in feature sharedness play an important role for distinguishing abstract from concrete concepts, other dimensions, such as effector relatedness might be additionally important. Furthermore, even in the domain of concrete concepts alone, non-prototypical representatives of a category may not share the entire spectrum of what may appear to be the set of fully-shared features (for discussion, see, for example, Pulvermüller, 2018a).

For simulating processes and representations underlying concrete and abstract concepts in the human brain, we here use a model of both peri-sylvian language areas along with areas further away from the sylvian fissure, including dorsal motor and planning related frontal areas as well as ventral visual perceptually related visual areas in temporal and occipital lobe. The model has previously been applied to study processes underlying learning of words with concrete meanings, including action- and object-related concepts (Garagnani & Pulvermüller, 2016; Tomasello et al., 2017, 2018, 2019). This model incorporates a range of neuroanatomical and physiological properties known to be important for sensorimotor, conceptual and language processing in the human brain, along with a mechanisms for unsupervised Hebbian learning (see “Methods”).

We use this ‘brain-constrained’ model of relevant cortical areas and their connectivity to investigate putative neurobiological mechanisms of concept formation. In addition, we will highlight any changes in the emerging conceptual model representations as a consequence of the highlighted structural difference in conceptual structure between concrete and abstract concepts, i.e. full feature overlap vs family resemblance, aiming at characterizing putative differences in the neurobiological correlates of these concept types. The results will be considered in light of experimental findings revealed by behavioural, neurophysiological and neuroimaging studies.

## Methods

Building on earlier modelling work (Tomasello et al., 2018, 2019), we used a neuroanatomically grounded, neurophysiologically plausible computational model with spiking neurons and 12 model areas representing visual and motor as well as auditory and articulatory areas in frontal, temporal and occipital cortices that are known to be important for processing words and their meaning.

### Model architecture^2^

We adopted a model architecture constrained by neurobiological information and previously applied to explore neural mechanisms of semantic learning (Tomasello et al., 2017, 2018, 2019). The following brain constraints were applied to the model (Pulvermüller et al., 2021):

(i) Neurophysiological dynamics of spiking pyramidal cells including temporal integration (summation) of inputs, threshold-based probabilistic spiking, and adaptation (Connors et al., 1982; Matthews, 2001) were implemented (following Tomasello et al., 2018);
(ii) Synaptic weights were modified by way of unsupervised Hebbian-type learning, including both long-term potentiation (LTP) and long-term depression (LTD) (Artola & Singer, 1993) (following Garagnani et al., 2008);
(iii) Global and local activity regulation (Braitenberg, 1978; Yuille & Geiger, 2003) and control were realized by area-specific and local inhibition (following Knoblauch & Palm, 2002);
(iv) 12 areas commonly distinguished in inferior and dorsolateral frontal, superior temporal and ventral temporal and occipital cortex were modelled (following Garagnani & Pulvermüller, 2016);
(v) Within-area connectivity included local excitatory and inhibitory connections (see also (iii)) excitatory connections were sparse, random and initially weak, exhibiting a neighbourhood bias towards close-by links (Braitenberg & Schüz, 1998; Kaas, 1997) (following Garagnani et al., 2008);
(vi) Between-area connectivity was implemented in accordance with neuroanatomical studies (see Table 1, following Tomasello et al., 2018) and following general anatomical principles (following Schomers et al., 2017; Tomasello et al., 2017);
(vii) Inherent baseline noise (white noise) was constantly present in all neurons of all areas during learning and while recording the network response to learnt patterns. In addition, peri-sylvian areas not receiving a specific pattern as input during learning received further uncorrelated white noise activation to simulate variable inputs (following Garagnani & Pulvermüller, 2016; Tomasello et al., 2019).

Further details about the implementation, including the equations implemented in the simulation software used, are provided in the Appendix.

### Simulated brain areas and their connectivity structure^3^

The spiking network model mimicked 12 different cortical areas with area-intrinsic connections and mutual connections between them. Note that we refer to model brain areas using an asterisk (e.g. *V1). Six areas were modelled for the left peri-sylvian language cortex including the primary auditory cortex (*A1), auditory belt (*AB) and modality-general parabelt areas (*PB) constituting the auditory system and the inferior part of primary motor cortex (*M1_i_), inferior premotor (*PM_i_) and multimodal prefrontal motor cortex (*PF_i_) representing the articulatory system (i.e. inferior face-motor areas). In addition, six extra-sylvian areas were modelled including the primary visual cortex (V1), temporo-occipital (TO) and anterior–temporal areas (AT) for the ventral visual system and the dorsolateral fronto-central motor (*M1_L_), premotor (*PM_L_) and prefrontal cortices (*PF_L_) for the dorsolateral action system.

**Fig. 2:**
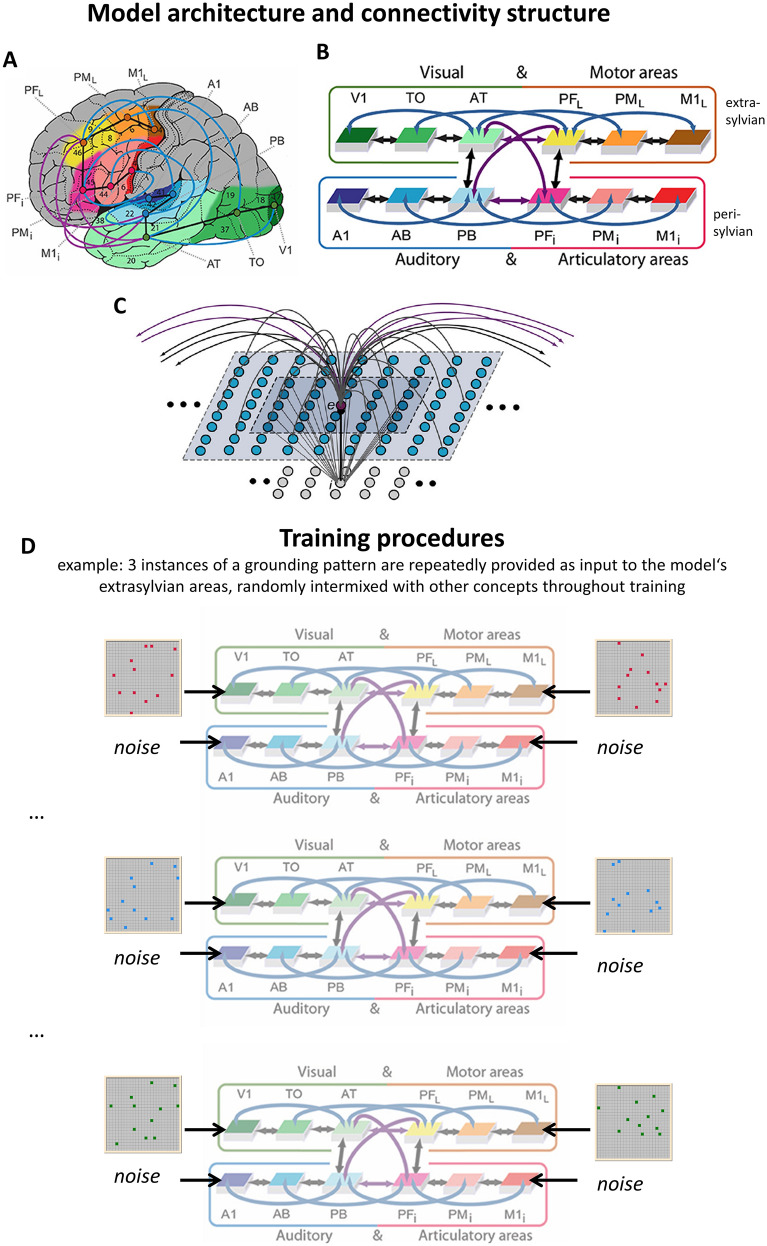
**A** Structure and connectivity of the neural network model. 12 brain areas were modelled in total, including areas in frontal, temporal, and occipital cortex. Peri-sylvian areas comprise an inferior–frontal articulatory (red colors) and a superior temporal auditory system (blue colors) and extra-sylvian areas comprise a lateral dorsal hand-motor system (yellow/brown) and a visual ‘what’ stream of object processing (green). Numbers refer to Brodmann Areas (BAs) and the arrows represent long distance cortico-cortical connections as documented by neuroanatomical studies (see Table 2 for neuroanatomical evidence). **B** Schematic depiction of the brain areas modelled (using the same coloring for different brain areas as in panel A), along with their connectivity structure. The different colors of arrows (black, blue, purple) stand for “next-neighbour” connections linking cortically adjacent areas within each system (black arrows) and “jumping links” between nonadjacent cortical areas within each system (blue links) as well as “long distance links” between pairs of multimodal areas PB, PF_i_, AT and PF_L_ (purple links). **C** An example of a single excitatory cell (labelled ‘e’; purple) and its micro-connectivity structure is shown. Within-area excitatory links (in grey) to and from cell e are limited to a local (19 × 19) neighbourhood of other neural elements (blue cells, light grey shaded area). Lateral inhibition between e and neighbouring excitatory elements is realized as follows: the underlying cell i inhibits e in proportion to the total excitatory input it receives from the 5 × 5 neighbourhood (dark grey shaded area); using analogous connections (not depicted), e inhibits all of its neighbours. Connections to other adjacent areas (black arrows) and non-adjacent areas (purple arrows) are also shown. **D** Training procedures are illustrated with the example of 3 related grounding patterns belonging to the same concept. On a given training trial, the motor and sensory component of a grounding pattern was provided as input to *V1 and *M1_L_, respectively, i.e. 12 neurons were activated in each area for 16 time steps. Peri-sylvian areas (*A1 and *M1_i_) always received uncorrelated noise as input. Note that during training, the 30 different grounding patterns belonging to 10 concepts were always randomly intermixed (indicated by the three dots between successive grounding patterns of the same concept). Panel A, B, parts of panel D and part of the descriptions have been adapted and modified from Tomasello et al., (2019) and panel C has been adapted and modified from Garagnani et al., (2017), both published under a CC-BY license (https://creativecommons.org/licenses/by/4.0/)

The network’s between-area connectivity structure reflects existing anatomical pathways between corresponding cortical areas revealed by neuroanatomical studies using diffusion tensor and diffusion-weighted imaging (DTI/DWI) in humans and nonhuman primates that are discussed in detail in a previous study (Tomasello et al., 2018) and summarized in Table 1. In summary, these anatomical pathways were modelled between adjacent cortical areas within each of the 4 ‘streams’ (see black arrows in Fig. 2) and between all pairs of multimodal areas (*PB, *PF_i_, *AT and *PF_L_) through the long distance cortico-cortical connections (purple arrows). In addition, as a previous neurocomputational study (Schomers et al., 2017) demonstrated the importance of non-adjacent ‘jumping’ links for verbal short-term memory, such second-next-neighbour links (skipping one intermediate area) were also included within the superior and inferior temporal and the superior and inferior frontal processing streams (blue arrows).

### Concrete and abstract grounding patterns

Children may be able to learn object- and action-related concepts just by perceiving instances of these concepts and by recognizing the similarities between them (Bornstein & Mash, 2010). It is possible that, in this learning process, some inborn category information comes in, but we here adopt the weakest assumption, namely that the categorial structure of the encountered entities is sufficient for category building. Therefore, we created patterns aimed at representing object perception and action execution to be used for stimulation in visual and motor extra-sylvian brain areas (*V1 and *M1_L_) while allowing the model to ‘learn’, that is, to modify synaptic weights according to biological learning principles. This strategy is based on the assumption that, when children acquire concepts, they often (i) experience visual perceptual patterns of the referent (modelled as *V1 activation here) and/or (ii) carry out actions (Baldwin, 1995) (modelled as *M1_L_ activation). Note that unlike earlier simulations on object and action concepts with the same model architecture used here (Garagnani et al., 2017; Tomasello et al., 2018), we did not make any distinction between action- and visually-related components of meaning, but rather treated all concepts as containing both components, as many concepts—both concrete and abstract—might involve both components (see Harpaintner et al., 2020 for recent fMRI evidence and Kiefer & Harpaintner, 2020, for a recent review). As such, we take every grounding pattern to consist equally of sensory and motor components used as input in *V1 and *M1_L_ and the simulated concepts can therefore be assumed to be grounded in both perception and action knowledge. The concrete concept of HAMMER, for example, contains both visually-related semantic features (knowledge about what it looks like) and action-related semantic features (knowledge about what it feels like to use a hammer). However, this rationale is not restricted to concrete concepts. A similar argument can be applied to abstract concepts with a family–resemblance relationship, e.g. DEMOCRACY, which may contain visual/perceptual aspects (perceptions of elections, raised hands, voting ballots, parliament buildings etc.) and action aspects (action of casting a ballot, raising one’s hand to vote etc.). The patterns presented to *V1 and to *M1_L_ can be viewed as two sub-components (visual and motor, respectively) of a single sensorimotor pattern extending equally across *V1 and *M1_L_. Thus, in contrast to earlier studies (e.g. Garagnani & Pulvermüller, 2016; Tomasello et al., 2017) we here did not specifically investigate differences between processing of object and action meaning, for example. Rather, we treated all concepts (both concrete and abstract) as containing referent instances including both visual and motor information.

In order to model effects related to semantic category learning, we created ‘grounding sets’ of grounding patterns each thought to represent 1 object and/or action. For each grounding set representing one semantic concept/category, we created 3 grounding patterns, whereby triplets of patterns showed different similarity structures for concrete and abstract concepts, exhibiting either full sharing of neuronal elements or family resemblance. There were 10 concepts per semantic category (abstract/concrete) and thus 30 instances of grounding patterns overall for each semantic category type. Based on the learning of the 3 related grounding patterns, we expected the model to learn and build representations of the 3 object/action instances (which the 3 grounding patterns stand for) and, crucially, a representation of the generalized semantic concepts themselves, either concrete or abstract.

Each grounding pattern consisted of 12 ‘active’ cells in *V1 and 12 *M1_L_ each (i.e. 12 ‘active’ out of the possible 625 neurons per area). Between the different concepts, there was never any overlap in the neurons making up grounding patterns and different models were built for concrete and abstract simulations (i.e. each individual model either received concrete or abstract grounding patterns, but never both types mixed in the same model). An example of 3 grounding patterns and their similarity structures is given in Fig. 1 and follows the idea outlined in the introduction that concrete concepts have feature overlap neurons which all instances of a grounding pattern representing a concept have in common (top panel). In contrast, for abstract concepts, there were no neurons common to all three instances, only neurons that 2 out of 3 instances had in common, i.e. pairwise shared neurons resulting in a family–resemblance structure (bottom panel). In addition to shared neurons, which we also call semantic neurons, both concrete and abstract concepts also had unique neurons only occurring in one grounding pattern. These can be thought to represent perceptual or action features that are not essential for defining the concept, but rather individual variations of instances of a concept (e.g. in the case of a concrete concept like HAMMER, specific colors or shapes of individual hammers that constitute some variation within the category but are not essential features). Therefore, we do not consider these ‘idiosyncratic neurons’ semantic or conceptual in nature.

For the purpose of the present simulations, we had to quantify the number and relationship of specific perception/action-related and shared semantic neurons for each concept type. Specific numbers of unique and shared neurons were chosen such that abstract and concrete concepts were matched both on the number of individual neurons per grounding pattern (12) and the number of distinct neurons occurring across the entire grounding set (24). Specifically, for concrete concepts there were 6 shared neurons (shared by all three grounding patterns) and 6 unique neurons per grounding pattern (6 + 3*6 = 24 distinct neurons); for abstract concepts, there were 4 pairwise overlapping neurons in 3 pair constellations of instances (i1·i2, i1·i3, i2·i3) and 4 unique neurons per instance (4*3 + 4*3 = 24 distinct neurons; for an illustration, see Fig. 1, bottom). Note that the matching in this respect means that concrete concepts’ grounding sets had fewer shared input pattern neurons in total (6) than abstract concepts (12). However, when each grounding pattern was activated once, the 6 concrete semantic feature neurons were activated 3 times each (18 activations) and the 12 abstract semantic feature neurons twice (24 activations overall). There were also more unique neurons for concrete than for abstract concepts in a grounding set (18 vs. 12). We note that these differences may lead to biases in the results, which we will address in the Discussion. Still, the matched conceptual structure implementations will enable us to draw careful conclusions on the distribution of unique and shared semantic neurons for each concept type.

### Training procedures

We ran a total of 12 instantiations of the model for each semantic type, comparable to running 12 human participants in an experiment, each with identical training patterns and procedures. To implement the equivalent of some random variation as would be present across individual human participants, we randomized for each model all synaptic links (and corresponding weights) between cells in connected areas (and within areas) before training (model initialization). The same set of initial randomized synaptic links and weights was then used to train a model with concrete patterns and with abstract patterns, but in separate model instances. Separate instantiations were used for the learning of concrete and abstract concepts to avoid interference between the two types of conceptual representations. Due to the shared initial randomized synaptic links (and in spite of the different networks for conceptual types), this amounts to a “within-subject” design, with each of the 12 model instantiation pairs representing one “subject”.

Each training trial consisted of randomly choosing one of the 30 sensorimotor patterns (consisting of 12 ‘active’ neurons per area, described in detail above) and presenting it as input to extra-sylvian primary areas (*V1 and *M1_L_) continuously for 16 time steps. In contrast to earlier studies (Garagnani & Pulvermüller, 2016; Tomasello et al., 2018), we did not intend to study effects of associating perceptuo-motor patterns with ‘word form’ pattern in peri-sylvian areas here, because it is sometimes assumed that concepts are learned before these are linked to language (Akhtar & Tomasello, 1996). Therefore, during conceptual learning, no correlated input was given to the language part of the model, the peri-sylvian primary areas *A1 and *M1_i_; instead, uncorrelated white noise stimulation was applied to these at all times, assuming that acoustic inputs and articulatory activity are unrelated to the conceptual patterns. The absence of input patterns to peri-sylvian areas (which would reflect “linguistic labels”) was a deliberate choice, as the scope of the present simulations was to map the similarity structure of the instances of concrete and abstract concepts and observe the consequences within a brain-constrained neural architecture. We are aware of the fact that after conceptual learning, linguistic learning (e.g. by always associating the variable conceptual instances with identical “verbal labels” in peri-sylvian areas) may add to and refine any neurobiological representations formed; this issue was outside the scope of the present work, but is currently being followed up (see “Discussion”).

To avoid possible contamination between successively presented stimulus patterns, an interstimulus interval (ISI) followed each pattern presentation. This ISI lasted until global inhibition in areas *A1 and *PB had returned below a specific threshold so that network activity had returned to a baseline value to prevent one trial from affecting the next one. During these ISIs the only input to the network was baseline white noise, simulating the spontaneous baseline neuronal firing observed in real neurons. Instead of stimulus patterns, white noise was also presented as input to all primary model areas (*V1, *M1_L_, *A1, *M1_i_) during ISIs. Training continued until 4000 repetitions of each instance of a pattern had occurred, i.e. 12,000 repetitions per concept.

### Testing procedures

**Fig. 3:**
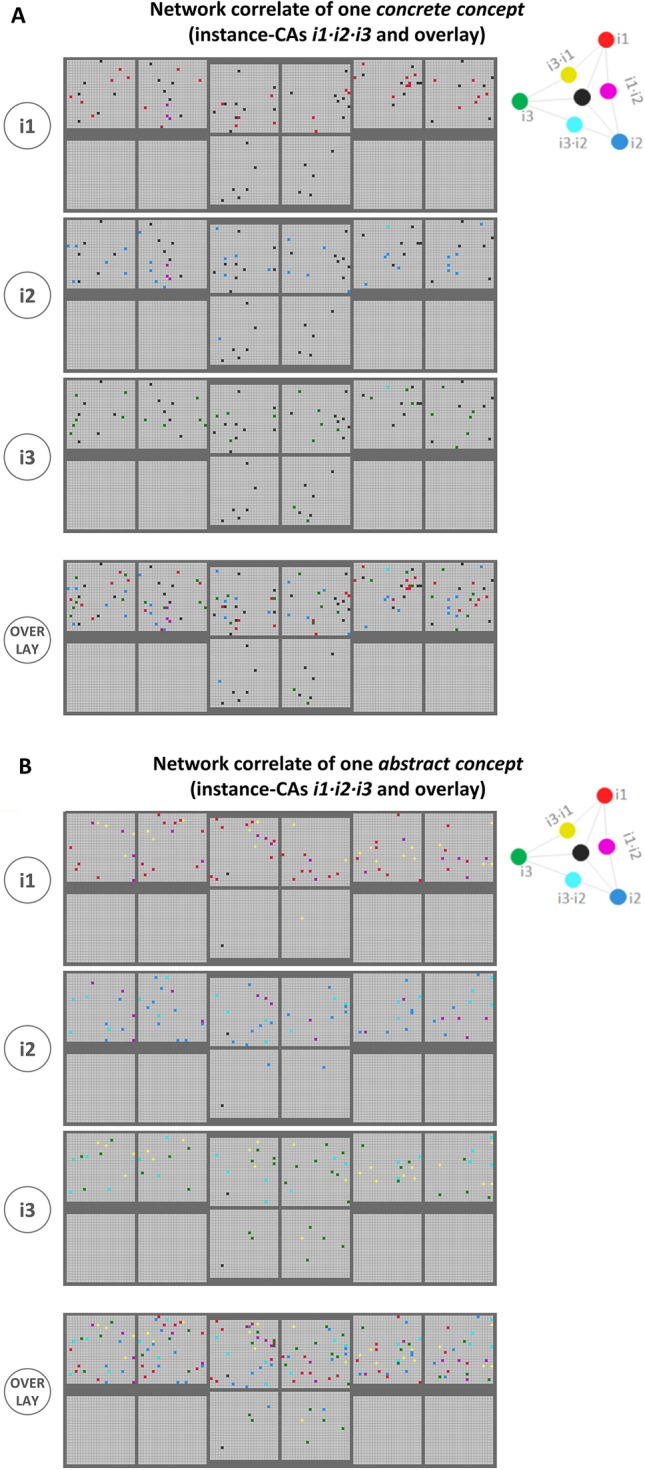
Examples of the network correlates of a concrete (top panel, **A**) and an abstract concept (bottom panel, **B**). For each type of concept, the neural correlates (cell assemblies, CAs) of each of the 3 instances (instance-CAs, top 3 rows) as well as an overlay of the entire concept-CA (bottom row) are shown. The 12 areas depicted are arranged in the same way as the model areas depicted in Fig. 2B (i.e. extra-sylvian areas on top, peri-sylvian areas on the bottom). The 4 central “hub” areas are shown as closer together because these areas are particularly strongly linked to each other (see Fig. 2A). Note that active neurons in the primary extra-sylvian areas (top left, *V1; top right, *M1_L_) are simply a consequence of the input of the grounding patterns during testing, whereas neurons in the other areas reflect the activation of CAs that emerged as a consequence of learning. The bottom part of each panel shows the overlay map with neurons being part of more than 1 CA (additive color mixing; neurons included in all 3 CAs in black). We assume that these non-unique neurons shared between more than one instance representations are key to the representation of a concept (see also Fig. 1). While this figure shows only one specific example of a concrete and abstract concept each, an interactive version allowing to view the full data set is available at https://osf.io/cmhx6/

After learning, a testing phase was implemented to examine the result of learning and to assess any representations of concrete and abstract concepts that may have emerged. To this end, each of the 30 trained sensorimotor grounding patterns were again presented to the extra-sylvian primary areas, *V1 and *M1_L_, recording the resulting instance CA (activated neurons in response to a single grounding pattern). In a second step, the resulting CAs were also analyzed with special attention to cell assembly overlap across the 3 related instance CAs (see Fig. 3 for specific examples for a concrete and abstract concept and the online version of Fig. 3 for the full data).

Prior to the presentation of each pattern, a global network reset was carried out, upon which the membrane potential of all excitatory and inhibitory cells was set to 0, to ensure that neuronal activity of a previously presented pattern did not affect results. Subsequently, each sensorimotor grounding pattern was presented for 2 time steps to extra-sylvian areas *V1 and *M1_L_ and network responses were recorded during stimulation and the subsequent 28 time steps (30 time steps total). During the 2 time steps of pattern presentation, no baseline noise was present in any area; during the subsequent 28 time steps of the recording phase, baseline noise stimulation was present in all model areas again, as during training. However, in contrast to the training phase, no uncorrelated white noise was given as input to the peri-sylvian areas (*A1, *M1_i_) during testing.

### Data analysis

#### Cell assembly circuit definition

To identify the neurons making up the distributed cell assembly (CA) circuits that had formed across model areas in response to each of the 30 grounding patterns, previously established procedures (Garagnani & Pulvermüller, 2016; Garagnani et al., 2017; Schomers et al., 2017) were applied. An excitatory neuron (or e-cell) was considered to be part of the CA circuit of a grounding pattern if and only if, on at least two time steps, its firing rate exceeded 75% of the firing rate of the maximally responsive cell in a given area in response to that pattern (provided the maximally responsive cell’s firing rate was at least 0.01, to avoid spurious results when the overall activity in an area was close to zero). As during training, we only used single grounding patterns at a time as input in the test phase, i.e. the response of the model to a previously-learnt pattern was recorded on a per-instance basis.

#### Sharedness calculation

To obtain clues on the network-internal correlates of conceptual processing, we focused, in a second step, on the analysis of the overlap structure of grounding pattern CAs. For each model area, neurons were classified according to whether they were activated by just 1 grounding patterns or whether they responded to 2 or 3, thus being part of the pair- or triple-wise shared overlap of grounding CAs. The shared or semantic neurons will be interpreted in the context of concrete and abstract concept representations. Note that the overlap structure of grounding patterns fed into the network already enforced specific overlaps between the cell assemblies forming in the network, but, strictly speaking, only the stimulated primary areas were directly influenced by this. How the stimulation patterns and their similarity structure influenced the similarity structure of the learnt cell assemblies expanding throughout the model network was a central question.

### Statistical analysis

To statistically test for the presence of significant differences in the CA circuit sizes and distributions of shared CA neurons across the model areas, we performed a repeated-measures 4-way analysis of variance (ANOVA) with the factors SemanticType (two levels: Concrete vs. Abstract) and the topographical variables PeriExtra (two levels: Peri-sylvian = {*A1, *AB, *PB, *M1_i_, *PM_i_, *PF_i_}, Extra-sylvian cortex = {*V1, *TO, *AT, *M1_L_, *PM_L_, *PF_L_}), Tempo(ral)Frontal (TempFront) (2 levels: temporal areas = {*A1, *AB, *PB, *V1, *TO, *AT}, frontal areas = {*M1_L_, *PM_L_, *PF_L_, *M1_i_, *PM_i_, *PF_i_}) and Centrality (three levels: Primary = {*A1, *V1, *M1_L_, *M1_i_}, Secondary = {*TO, *AB, *PM_L_, *PM_i_} and Central = {*PB, *AT, *PF_L_, *PF_i_} areas). Note that although abstract and concrete models were run separately, due to the shared initial randomized synaptic links between pairs of abstract and concrete models (see “Training procedures”), we treated SemanticType as a “within-subject” factor.

All data processing, statistical analyses and figure creation was performed using Python (version 3.7), numpy (version 1.19.2; Harris et al., 2020), pandas (version 1.1.5; McKinney, 2010), matplotlib (version 3.3.2; Hunter, 2007), seaborn (version 0.11.0; Waskom, 2021), scipy (version 1.5.2; Virtanen et al., 2020) and statsmodels (version 0.12.1; Seabold & Perktold, 2010). The significance threshold was adjusted to a conservative critical p of 0.01.

## Results

After the grounding patterns had been presented repeatedly (4000 presentations per pattern) while Hebbian correlation learning was effective, the network had developed strongly connected neuron ensembles or cell assemblies (CAs) for each grounding pattern. Figure 3 shows, for illustrative purposes, the neural correlates (cell assemblies, CAs) for one specific example each of a concrete concept (Fig. 3A) and an abstract concept (Fig. 3B). Note that the full CA data for all 10 concrete and 10 abstract concepts can be interactively viewed in an online version of the figure at https://osf.io/cmhx6/. In each panel of Fig. 3, the top three rows show the instance-CAs, that is, the neural correlate of one individual sensorimotor pattern, whereas the bottom row shows an “overlay map” of the three related instance-CAs. Neurons present in only one instance-CA are shown in one of the main colors (blue, green, red). These CAs were scattered across the entire extra-sylvian part of the network architecture and even extended into connector hub peri-sylvian areas (*PF_i_, *PB). As argued in the Introduction, we consider features that are shared between instances of a concept to be semantic or conceptual. Therefore, at the neuronal level, we asked which neurons can represent such shared semantic features. These are the neurons included in more than one instance CA. The bottom parts of the left and right panels of Fig. 3 show these ‘shared’ neurons in colors resulting from additive color mixing (cyan = blue + green, magenta = blue + red, yellow = red + green, white = blue + red + green); neurons that are part of all 3 instance-CAs are colored black. It can be seen that for concrete concepts, the triple-shared neurons are distributed across all extra-sylvian areas, even perhaps with a tendency to increase in number towards the middle of the network. In contrast, the grounding sets of abstract concepts yielded very few triple-overlap neurons (in black), which is unsurprising, because the grounding patterns did not include them either. However, it may appear that, for abstract concepts, there are fewer shared conceptual neurons towards the middle of the network as compared with the primary areas. Further analyses focused on quantitative analysis of the distribution of unique and conceptual neurons across network areas.

### Quantitative analysis of instance-CAs (Fig. 4)

**Fig. 4:**
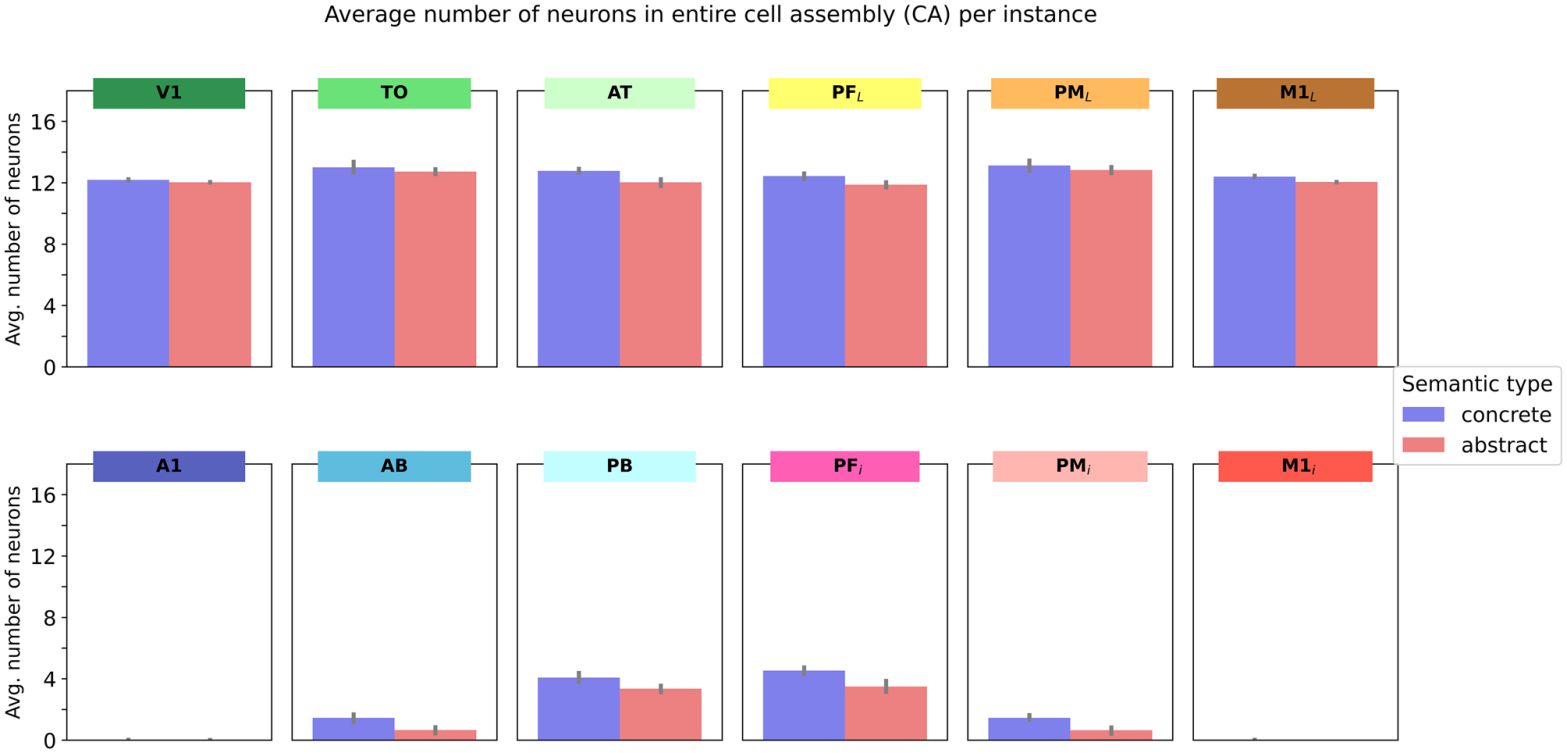
Average number of neurons in instance-CAs (cell assemblies activated in all 12 model areas in response to individual grounding patterns as input to *V1 and *M1_L_ in the testing phase). Error bars show 95% confidence intervals

In a first step, we analyzed the sizes of the emerging cell assembly (CA) sizes in extra-sylvian model areas in response to stimulating all the learnt grounding patterns after training, calculating the number of CA neurons separately for each instance of a concept (instance CAs). Figure 4 shows the average number of neurons in instance CAs. As the 4-way repeated measures ANOVA (SemanticType(2) × PeriExtra(2) × TempFront(2) × Centrality(3)) revealed a significant 4-way interaction (*F*(2,22) = 8.5, *p* = 0.0019), we performed further ANOVAs for extra- and peri-sylvian areas separately. For the ANOVA for peri-sylvian areas, we excluded the primary areas *A1 and *M1_L_ as these contained virtually no activated neurons at all (average CA sizes 0 or 0.01). The 3-way ANOVA (SemanticType(2) × TempFront(2) × Centrality(2 levels only; secondary vs. central) showed a significant main effect of SemanticType (*F*(1,11) = 120, p < 0.0001) and Centrality (*F*(1,11) = 1973, *p* < 0.0001), but no interactions.

The 3-way ANOVA (SemanticType(2) × TempFront(2) × Centrality(3)) on the extra-sylvian CAs showed main effects of SemanticType (*F*(1,11) = 14.8, *p* = 0.0027) and Centrality (*F*(2,22) = 103, *p* < 0.0001), but no significant interactions. Bonferroni-corrected paired *t* tests for the three levels of Centrality (3 comparisons: primary vs. secondary; secondary vs. central; primary vs. central, critical *p* = 0.0033) showed that overall CA sizes were not significantly different between primary and central areas (primary areas: *m* = 12.16, central areas *m* = 12.27, *p* = 0.0118), but significantly larger in secondary areas (*m* = 12.92), both compared to primary (*p* < 0.0001) and central areas (*p* < 0.0001). Numerical inspection showed that although this peak in CA sizes was found in secondary areas for both concrete and abstract concepts, CA sizes in central areas decreased again compared to those in secondary areas more strongly for abstract than concrete concepts, although, as noted above, this interaction between SemanticType and Centrality was not significant. In summary, we see the typical overall “belly shape” (inverse *U* shape) of cell assemblies (more neurons in secondary and central areas) which has been shown in several previous simulation studies (Garagnani & Pulvermüller, 2016; Tomasello et al., 2017, 2018), and numerically, a slightly more pronounced “belly shape” for concrete than for abstract concepts, although these effects were small and nonsignificant. We note that further investigations about this shape feature were done in the context of analyzing CAs in the context of the three related instance-CAs (see next section) rather than as isolated instances.

### Neural correlates of concepts (Figs. 5, 6, 7, 8)

**Fig. 5:**
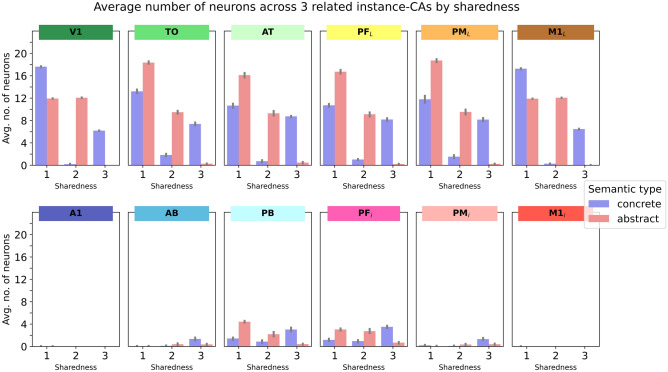
No of neurons involved in the processing of concrete and abstract concepts. Instance-specific neurons activated in response to only single grounding pattern have “sharedness” (across the CA representing the entire concept) of ‘1’. Semantic neurons activated in response to two or three different grounding patterns of one concept are labelled ‘2’ or ‘3’. The diagram shows how many unique (sharedness 1) and semantic (sharedness 2 or 3) neurons are present in the different areas of the network. Note that neuron distribution in the stimulated primary areas *V1 and *M1_L_ is simply a result of the pre-defined stimulation patterns (Fig. 1), but that the number of specific and shared semantic neurons across all other areas is the result of the learning process and changed in different ways for concrete and abstract concepts. Error bars show 95% confidence intervals

**Fig. 6:**
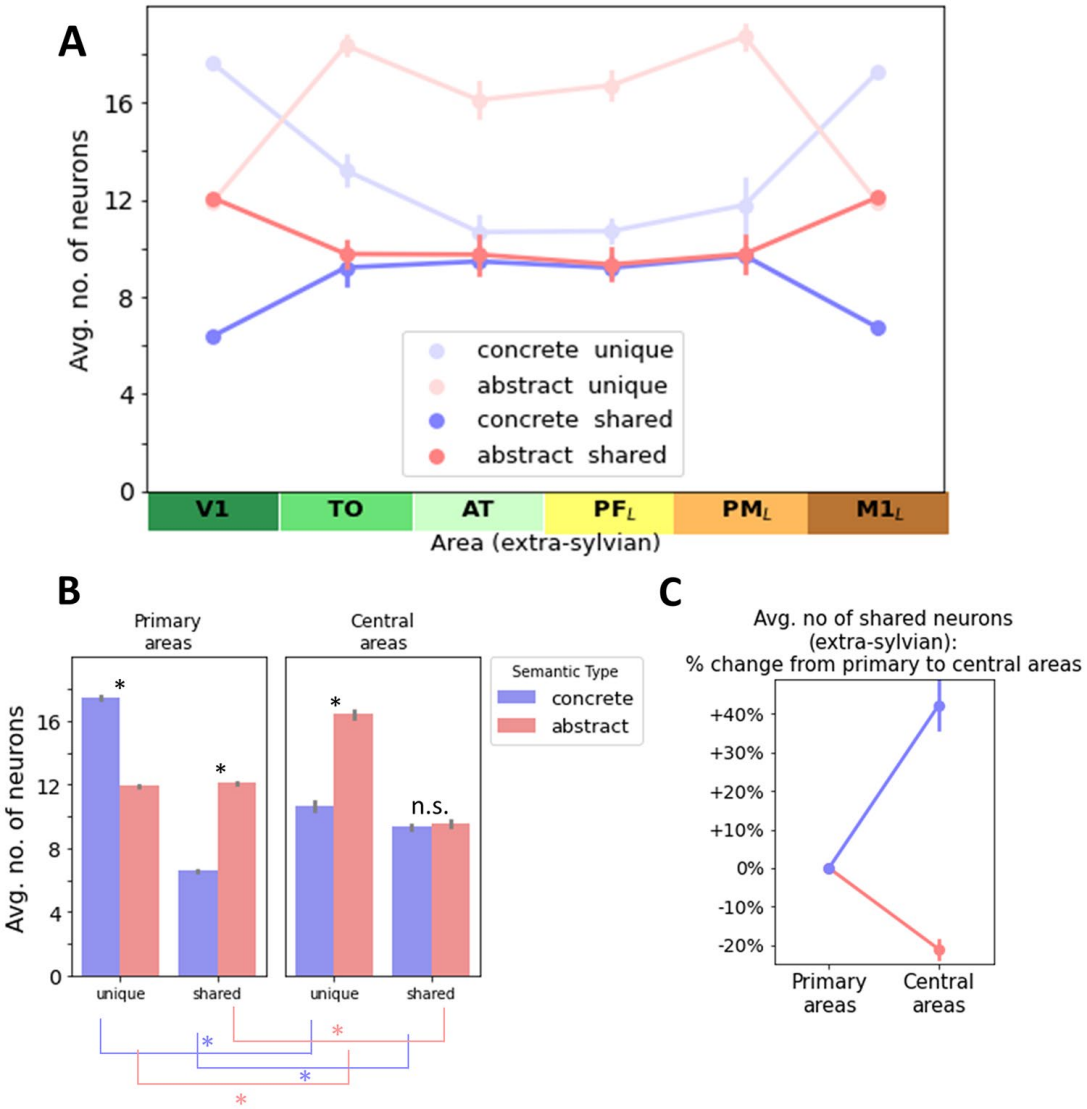
Distribution of unique instance specific and shared semantic neurons across the model’s 6 extra-sylvian areas. ‘Unique neurons’ are specific to one instance representation (or cell assembly). ‘Shared neurons’ are part of more than one instance representations/cell assembly. **A** The numbers of unique and shared neurons are shown per areas for concrete and abstract concepts. **B** The same data as in panel (A) but collapsed into primary (M1_L_, V1) and central areas (AT, PF_L_). Significant interactions (see main text for details) showed that for unique neurons, the relative pattern flips when moving from primary to central model areas. In contrast, for shared neurons, although abstract concepts start out with more shared neurons than concrete ones, they result in similar numbers in central areas. **C** To further illustrate in particular the divergence of the change in shared neurons from primary to central areas, we also calculated this change in percentage, indicating that the shared-by-all neurons present in grounding sets of concrete concepts lead to an increase of shared neurons in the resulting concept CAs whereas for abstract concepts, the pairwise shared neurons in grounding patterns decrease in the instance CAs in central semantic hub areas. See main text for a discussion of the role of unique and shared neurons in the neuronal concept representations of abstract and concrete concepts. Error bars show 95% confidence intervals

**Fig. 7:**
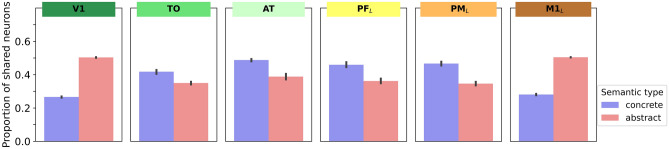
Proportion of shared neurons divided by the total number of neurons in instance-related cell assembly neurons across extra-sylvian areas. For concrete concepts, a “belly” shape (inverse *U*-shape) can be seen such that this proportion is higher in secondary and central areas than in primary areas which receive input. The converse, a “slim” shape (*U*-shape) is seen for abstract concepts, where proportion of shared neurons decreases in secondary and central areas. Error bars show 95% confidence intervals

**Fig. 8:**
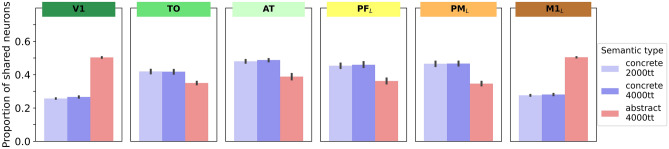
Same data as in Fig. 7 (proportion of shared neurons divided by the total number of neurons in instance-related cell assembly neurons), but with an additional control condition included (concrete concepts with only 2000 training trials (tt) instead of 4000). As outlined in the Methods and Discussion section, due to the fact that shared neurons occur in 3 out of 3 grounding patterns for concrete concepts, but only 2 out of 3 for abstract concepts, the shared neurons of concrete concepts are presented more often when comparing data for 4000 training trials for both semantic categories. These data show that even when reducing training trials for concrete concepts to 2000, thus actually giving the shared neurons in concrete concepts a slight disadvantage (overcompensating for this confound), the ‘belly’ vs. ‘slim’ shape for concrete and abstract concepts is still present, ruling out that semantic slimness effects are a result of the differential frequency of shared neuron presentations alone. Error bars show 95% confidence intervals

It was crucial to distinguish those parts of the representations of objects, actions and scenes that relate to specific sensory or motor features of these entities from those properties that reflect general conceptual features common to all or at least a subsection of the instances of a concept. Therefore, further analyses focused on the distinction between ‘unique’ instance-specific neurons thought to represent specific sensorimotor but not conceptual properties and ‘shared’ neurons thought to index features common to conceptual instances, which are therefore represent conceptual properties. Since, in our present simulations, each concept was learnt on the basis of 3 grounding patterns including both specific and shared neurons, we investigated the distribution of specific and shared neurons between concrete and abstract concepts. To this end, we quantified for each neuron activated by the instances of a concept whether it was unique (occurring in one instance only) or shared with at least one other instance (neuron counts by sharedness—Fig. 5). In this approach, sharedness is an index of conceptual status (rather than single-exemplar relatedness).

We restricted further analysis to the extra-sylvian areas and considered sharedness as a binary factor only (unique—occurrence in 1 instance-CA, shared—occurrence in 2 or 3 instance-CAs) (Fig. 6A). For statistical analysis, we also considered centrality as a binary factor, distinguishing primary and central connector hub areas only. The secondary areas were omitted because they frequently occupied an intermediary role, which complicated the analysis without producing additional relevant results.

A 2 × 2 × 2 ANOVA with factors SemanticType, Centrality and Sharedness revealed significant main effects of SemanticType (*F*(1,11) = 1646, *p* < 0.0001), Centrality (*F*(1,11) = 227, *p* < 0.0001) and Sharedness (*F*(1,11) = 5569, *p* < 0.0001) as well as significant interactions between SemanticType and Centrality (*F*(1,11) = 1646, *p* < 0.0001), SemanticType and Sharedness (*F*(1,11) = 260, *p* < 0.0001), Centrality and Sharedness (*F*(1,11) = 80.7, *p* < 0.0001) and a significant three-way interaction (*F*(1,11) = 2518, *p* < 0.0001). Bonferroni-corrected paired *t* tests (8 comparisons, critical *p* = 0.00125) between semantic types showed that the number of shared neurons in central areas was not significantly different between abstract and concrete concept representations (*p* = 0.16), whereas, in primary areas, a significant difference was found (*p* < 0.0001) (see Fig. 6B). Comparisons on the difference in number of neurons for primary vs. central areas for the same combination of SemanticType and Sharedness were all significant (*p* < 0.0001) (see colored bars below Fig. 6B).

To further pin down the relevant semantic differences, we focused on the relative change in shared (and thus conceptual) neurons only from primary areas to central areas, setting the number in primary areas as baseline (0) and expressing the number in central areas in % change from this baseline (Fig. 6C). Bonferroni-corrected *t* tests revealed that the number of shared conceptual neurons was significantly higher in central areas when compared with baseline (primary areas) for concrete concepts (+ 42.3%, SEM = 1.89%, *p* < 0.0001), whereas for abstract ones, there was a significant decrease (-21.1%, SEM = 0.74%, *p* < 0.0001).

An alternative way to summarize this differing distribution of unique vs. shared neurons is shown in Fig. 7, which is the proportion of shared neurons in each triplet of related instance CAs. Looking at the distribution of shared neurons across extra-sylvian areas from a distance, it seems that concrete concept processing involves a “belly” of relatively high shared neuron densities towards the middle of the network architecture (inverse *U* shape). In contrast, abstract concept representations have fewer shared neurons towards the middle of the network, thus leading to a “slim” distribution (*U* shape). An ANOVA with factors SemanticType(2) and Centrality(3) revealed a main effect of SemanticType (*F*(1,11) = 844, *p* < 0.0001), Centrality (*F*(2,22) = 160, *p* < 0.0001) and a significant interaction (*F*(2,22) = 211, *p* < 0.0001). Thus, in the CA circuits that developed in more central network layers, the proportion of shared neurons for abstract concepts was lower than the proportion in the grounding sets supplied as training input, indicating that the emerging CA circuits rely relatively more strongly on unique features. The converse was observed for concrete concepts: although their grounding sets contained more unique features than shared ones, the relative contribution of these unique features decreased towards central network layers, thus statistically supporting their “belly shape”. This visual observation was confirmed by Bonferroni-corrected paired *t* tests (12 comparisons, critical *p* = 0.0008) in each area, which confirmed that in primary areas shared neuron proportion was higher for abstract than concrete concepts whereas the converse was true in the four secondary and central areas (all *p* < 0.0001).

The same Bonferroni-corrected paired *t* tests were also run comparing concrete concepts with only 2000 training trials (tt) to abstract concepts with 4000 training trials (Fig. 8) to address a possible confound related to the fact that the shared neurons of concrete concepts occur in 3 out of 3 grounding patterns, whereas those for abstract concepts occur only in 2 out of 3 (see Fig. 8 and “Discussion” section on “putative shortcomings” for more detail).

## Discussion

We used a neurobiologically constrained model of peri-sylvian and extra-sylvian cortex to simulate the putative brain mechanisms underlying conceptual category processing along with possible differences between concrete and abstract concepts. The simulations rest on the assumption that, at least in some cases, categories are learnt and grounded based on experiences of instances of a category, i.e. objects, actions and circumstances that fall under the category, and by mapping the similarity structure of these instances on neuronal matter. Different similarity structures were implemented for concrete and abstract categories, with the former sharing semantic features across all category instances and the latter exhibiting family resemblance with only partially shared features (see “Introduction” and Fig. 1). Taking the neuronal correlates of generally and partially shared features as the mechanistic basis of category representations, we found that the learning of category instances entailed the formation of neuronal category correlates which were distributed across all sensory and motor areas through which instance-related information was processed and extended into areas in the centre of the network where information from different modalities converge. These central and multimodal ‘connector hub’ areas even exhibited larger semantic/conceptual neuron numbers than the modality specific primary areas in the case of concrete concepts (thus showing a ‘belly’ shape). However, in the case of abstract concept representations, the central connector hub areas carried relatively fewer conceptual/semantic neurons (‘slim’ shape) (see Figs. 5 and 6).

The belly-like and slim shapes of conceptual representations of conceptual categories have functional implications. Cell assemblies with numerous and strongly interlinked neurons in their centre may function as a unit, as a closed system. If sufficiently stimulated, they will activate as a whole (ignition) and after ignition, activity may persist and reverberate in the circuit for some time; the term ‘cell assembly’ or ‘conceptual circuit’ can be applied in this case (Braitenberg, 1978; Pulvermüller & Garagnani, 2014; Pulvermüller et al., 2014). If only sparse and weak links are present in the centre of an ensemble, the functional unity of the ensemble is not guaranteed (Schomers et al., 2017). Therefore, it appears that conceptual grounding builds solid representations of concrete concepts, but not necessarily ones for abstract categories.

### Putative shortcomings

This result emerged from a study where concepts were mapped based on the similarities of their instances. No verbal labels were associated with the conceptual instances and hence no explicit feedback was given to the network about whether individual instances belonged to a specific category or not. Although concept learning can, in principle, take place even without verbal labels, as shown by evidence from infants (Behl-Chadha, 1996; Bomba & Siqueland, 1983; Quinn et al., 1993) and non-human animals (Cook & Smith, 2006; Smith et al., 2008; Zentall et al., 2008), humans typically learn concepts in conjunction with a verbal label. The lack of verbal information is an obvious shortcoming of this work and calls for further simulations where instances are co-presented with symbols. It may well be that the lack in ‘belliness’ and the possible functional deficit in abstract concept processing, which our results indicate, can be remediated by verbal label information, which we are currently investigating in a follow-up study. Still, we should insist that the current simulations are important because they set a baseline of conceptual mappings without additional information from labels which may be important for future investigation. Against these results, any conceptual learning including feedback can fruitfully be interpreted.

A possible confound in our results is that the belly-shape of concrete and slimness of abstract concepts representations could be a consequence of not properly matched numbers of activations of neurons and representations in our present simulations. Although for obtaining the main results, each grounding pattern was presented 4000 times, the number of activations of shared neurons differed between conceptual types. Each semantic neuron of abstract concepts was activated twice, thus resulting in 8000 activations overall, but 12,000 activations resulted for concrete conceptual neurons, which were part of all three conceptual instance representations. This imbalance could account in part for the slimness or ‘belliness’ of representations.

To address this putative limitation, we compared the proportion of shared neurons for the abstract models after 4000 training trials per grounding pattern (i.e. 8000 per shared neuron) with the concrete models with reduced learning, after only 2000 training trials per grounding pattern, thus resulting in 6000 activations per shared neuron. Despite the fact that this comparison overcompensates the ‘disadvantage’ of shared neuron activations when comparing abstract and concrete models of identical total training trials, we nonetheless observed the same pattern of results with almost identical results for the 2000 and 4000 training trial simulations of concrete concepts (see Fig. 8). Regardless of the number of learning trials, concrete concepts showed the same belly-shaped distribution of shared semantic neurons across areas, thus contrasting in the same way with the proportion of semantic neurons in abstract conceptual neuron distributions. In other words, the pattern of more ‘semantic’ neurons for concrete concepts in central areas persists even when the semantic neurons of concrete grounding patterns are activated less frequently than those of abstract grounding patterns, ruling out that the semantic slimness/belliness effects observed are a result of such a confound.

More generally speaking, we would like to note that for any neural network simulation, results are specific to the network type and features used and this also applies here. This caveat would equally apply to behavioural experiments, where typically many parameters of an experiment (such as size of stimuli or interstimulus intervals) are also fixed throughout an experiment. Although we explicitly addressed the possible confound of number of repetitions here, we cannot rule out that some of our results depend on specific values of other parameters or network features, as is always the case for neurocomputational results.

### The role of shared neurons

The shared neurons can be seen as reflecting semantic features (see “Introduction” and first paragraph of Results section) and our results show that only for concrete concepts do new semantic neurons arise in the central network areas. Interestingly, in the case of concrete concepts, central areas exhibit 42% more such shared neurons than present in primary areas, as imposed by the similarity structure of grounding patterns. In sharp contrast to this increase, the shared neurons of abstract concepts actually decreased by 21% in central areas compared to primary areas (Fig. 6B). The lack of any semantic overlap neurons shared across all pattern instances in the grounding sets for abstract concepts seems to present a severe impediment to developing neuronal representations that have a large proportion of shared neurons. It is noteworthy that this ‘disadvantage’ is present despite the fact that abstract concepts (compared to concrete ones) actually have more shared neurons in their grounding patterns, both at the level of an individual grounding pattern (8/12 vs. 6/12) as well as across the grounding set (12/24 vs. 6/24). The qualitative difference in sharedness (pairwise overlap for abstract concepts vs. semantic feature neurons for concrete concepts) might therefore be the decisive factor which prevents abstract concepts from developing a strong cell assembly based on shared neurons. In contrast, the concrete concept representations actually develop new shared neurons in central model areas despite starting out with fewer shared neurons in the grounding patterns than in those for abstract concepts. Considering that we get similar results even when comparing concrete models with fewer repetitions per shared neuron, this suggests that only the shared-by-all neurons present in the grounding sets of concrete concepts can form the basis of strong CAs, whereas family resemblance appears insufficient, even when given a relative advantage in terms of number of repetitions (see Fig. 8). Note, however, that we here did not systematically disentangle differences in qualitative and quantitative overlap, i.e. these two factors were confounded in our design and we therefore cannot state with certainty whether the effects observed here reflect family resemblance per se or rather degree of overlap without family resemblance. Nonetheless, as the two frequently come together, we believe that the present results provide a reasonable advance in understanding. As outlined in the Methods section, we here chose the specific numbers of overlap such that both semantic types were matched for total number of neurons per grounding pattern (12 per area) and total number of distinct neurons occurring in the entire grounding set (24 per area). Matching the absolute or relative number of shared neurons in grounding patterns between semantic types would have meant giving up this matching in terms of total number of neurons. Future investigations should further elucidate the relative influence of qualitative vs. quantitative differences in overlap structure on our results.

### The role of unique neurons

Given that to our knowledge this is the first neurocomputational study to investigate processes underlying perceptual concept formation for concrete and abstract family–resemblance concepts, we cannot, based on the present results alone, provide a definitive explanation for the observation that the number of unique neurons in secondary and central areas is so much lower for concrete concepts than for abstract concepts even though the grounding patterns in primary areas actually contain *more* unique neurons for concrete than abstract concepts. Further neural network experiments will be necessary to elucidate the mechanisms explaining this (somewhat paradoxical) dissociation. However, the following tentative explanation can be offered: concrete pattern triplets have neurons shared between all three instances (shared-by-all), which, because they are activated most frequently, become dominant within the neuronal population activated by the concept instances and therefore lead to a relative suppression of the unique neurons. One might say that a strong core of conceptual neurons emerges from each concrete category (pattern triplet). In contrast, for abstract pattern triplets, there are no neurons shared between all three instance representations. Hence there are no neurons in the emerging CA sufficiently active to exert suppression of the emergence of new unique neurons in deeper (more central) layers. Instead, the unique neurons can contribute relatively more strongly to CA activation. One implication of this suggestion is that representations of abstract concepts are more strongly reliant on the unique features, although it is unclear if this means that unique features are more important for abstract concepts per se or simply play a larger role in the emerging representation because of less ‘suppression’ due to shared-by-all neurons being almost (but not completely, see Fig. 5) absent. One could interpret the unique features as representing to some extent contextual features, i.e. each situation or context in which a concept is experienced is different and therefore each situation/context is associated with its own unique features. As such, the greater reliance on unique neurons observed in our simulations fits with assumptions immanent to context availability theory (Schwanenflugel et al., 1988, 1992) and related theories (Davis et al., 2020), supported by experimental evidence showing that processing abstract concepts is more dependent on relevant contexts (Schwanenflugel, 1991; Wilson-Mendenhall et al., 2013) than processing concrete concepts. One might argue that concrete concepts possess shared-by-all neurons which encode semantic features that are central to the meaning of the concept and are therefore relatively independent of context (but note that the representation of concrete concepts is by no means entirely independent of context, see Yee & Thompson-Schill, 2016 for review). In contrast, abstract concepts developed more unique neurons and fewer and only partially shared ones. We interpret this to mean that the semantic representation of an abstract concept is weaker and hence on its own less sufficient for understanding a concept. As such, our model fits with the observation that concrete concepts are relatively easier to understand on their own, whereas abstract concepts might require more contextual information to complement the information from the stored semantic representation in order to be fully understood. Note, however that the present simulation does not explicitly model ‘context’ and hence does not allow us to test the context dependency of abstract concepts directly, although this too could be a subject of future investigation.

Another way to think about this effect in terms of informational content is this: for concrete concepts, the unique features represent relatively "minor details" that differ between instances of the same concept. For example, in the case of the concept HAMMER, the length, material and color of the handle, shape and size of the head etc. would be such "minor details" represented in unique features. These “minor details” would all be important for distinguishing different kinds of hammers, but would not “make or break” the category membership, i.e. what makes an object a hammer does not depend on such “minor details”. As such, the unique features for concrete concepts do not influence the shared semantic features of the concept (which instead is represented in the shared-by-all semantic feature overlap neurons). In contrast, the unique features might play a much more integral role for defining abstract concepts because these do not have the strong shared-by-all neurons, only family resemblance shared-by-2 neurons. 

### Category differences in language acquisition

We now turn to a not yet fully understood fact known from language acquisition and neurocognitive research, which may be open to a mechanistic explanation suggested by the current results. It is well known that young children learn category names for concrete objects much earlier than abstract terms; action-related words seem to be learnt later than concrete object words, but still before the abstract ones (Au et al., 1994; Bassano, 2000; Bergelson & Swingley, 2013; Gentner & Boroditsky, 2001; Kauschke & Hofmeister, 2002). We note that most empirical evidence on this question is indirect, as it typically is based on findings that nouns are learned earlier than verbs as evidence for concrete words being learnt before abstract words, but this might be confounded (for review, see Vigliocco et al., 2011). Furthermore, even assuming that infants’ early words are predominantly concrete object words, it is unclear whether this really reflects greater difficulty in learning abstract words or the fact that caretakers tend to predominantly use object words. As a further caveat, considerable cross-linguistic variation appears to be present in the noun dominance in early language acquisition (e.g. Kauschke et al., 2007; Tardif et al., 1997) and to what extent (concrete) noun bias is a universal phenomenon or rather purely language specific is a topic of great debate (for review, see Waxman et al., 2013). Novel data (Setoh et al., 2021) suggests, however, that the early noun dominance is indeed a widespread phenomenon with cross-linguistic differences exerting only a minor effect. Assuming that priority of object words in early language acquisition is indeed a fundamental feature of human language learning, one reason for this priority of the concrete could lie in the teaching strategies of adults or the persistence of solid objects, which, in contrast to instances of abstract concepts, do not change in a fast, situation-dependent manner. However, it may also be that it is systematically more difficult to build abstract conceptual representations compared with concrete ones. One reason for this could lie in the different conceptual structures discussed in the present work (feature sharing vs family resemblance) and their neurocomputational implications studied by the simulations, in particular the relatively weaker neuronal representations of abstract concepts in central connector hub areas. As generally-shared semantic features are missing for abstract categories, conceptual learning may require not only more time but putatively additional qualitative factors enabling the formation of strongly connected concept representations. One possibility is that experiences with many more variable instances are required than for concrete concepts. A further possibility is that linguistic information (for example, associating concepts with verbal labels) plays an important role in abstract concept formation (Borghi & Zarcone, 2016; Dove, 2018; Dove et al., 2020; Lupyan & Clark, 2015; Waxman & Markow, 1995), where for the shared-feature concrete concepts no linguistic enhancer is required. However, we wish to stress that these suggestions call for more work, both at the experimental side and at the neural simulation end.

We should also emphasize that the late learning of abstract concepts can be explained by alternative approaches. Considering the proposal by Barsalou and Wiemer-Hastings (2005) that reference to internal states and processes is required for and plays a great role in abstract concept learning, it appears as plausible that such a process may just per se be more demanding than the reference to concrete objects accessible to the entire community. Even if one acknowledges that internal states can only be assessed and labeled if action-based criteria are available (see “Introduction”), such grounding of inner state concepts in action could imply a greater learning effort than the inter-linking of concrete objects with shared properties. Still, also in this framework, a mechanistic explanation is desirable and it may turn out that the structural difference between shared features and family resemblance plays a role here too.

### Future research needs

The reported simulations indicate that the role of unique features might differ between concrete and abstract concepts. These simulations build a causal chain from conceptual structure (feature sharing vs family resemblance) to neurobiological mechanisms, but as we have already mentioned, various aspects of this model are calling for further research. Here, we would like to highlight that we suggested that the neuronal representations of concrete concepts are somewhat more strongly represented, not only in terms of quantity of shared neurons but also in terms of function, as compared with abstract ones. To confirm this, further simulations are necessary targeting the dynamics of neuronal activation—in view of variable cognitive processes, including the spontaneous ‘coming to mind’ of a concept.

Regarding causality, we should also mention the following: the present simulation data alone show that different overlap structure in grounding patterns causes different overlap structure in cell assemblies, but does not allow us to draw any *causal* conclusions about the functional role that unique and shared neurons in the central layers of the model play in the activation of a concepts’ CA. One possible avenue for further investigation of this topic would be to leave out the unique neurons in the testing phase, i.e. train the model on shared and unique neurons (as we have done here), but then only stimulate with the subset of neurons that was shared and test how this reduced stimulation might differentially impair cell assembly activation for concrete and abstract concepts. Similarly, selective lesioning of shared or unique neurons in the deeper layers could be done to investigate the relative causal contribution of these neurons to the activation of a CA.

Finally, another important limitation is that our present modelling approach does not allow specifying at what level of specificity in the hierarchy we are modelling semantic categories and their members. However, this apparent limitation can also be seen as an advantage, as the results generalize to several levels of the conceptual hierarchy. Although we introduced our simulations as reflecting basic-level concepts with 3 object instances each, the hierarchy implemented in the model can also be interpreted to reflect a domain-level category and 3 basic level categories included in that domain, i.e. it could either be viewed as a basic-level category with three individual members (e.g. concept CHAIR with 3 instances of chairs differing in some details) or a domain-level category with different basic-level concepts making up that category (e.g. concept FURNITURE with 3 members CHAIR, SOFA and TABLE).

The way we model concepts as consisting of grounding patterns with equal numbers of static sensory and motor features is obviously also another important limitation that should be considered when interpreting our findings. Real concepts—both concrete and abstract—will likely differ in the relative contribution of sensory and/or motor features and findings from previous simulations (which were done for concrete concepts only) have indicated that topological differences in the cell assemblies exist for object vs. action words (Garagnani & Pulvermüller, 2016; Tomasello et al., 2017, 2018), leaving open the question of what would happen to abstract concepts which are grounded either purely in sensory or purely in motor features. Approaches that view the difference between abstract and concrete concepts in the relationship to internal states, such as emotions and affects, are therefore not covered by this approach. The reason for omitting this difference is our conviction that any ‘grounding in emotion’ is by necessity grounding in action, so that any emotional grounding in so-called ‘internal states’ is in fact realized as and based on action grounding (see e.g. Moseley & Pulvermüller, 2018). Because, in this perspective, abstract emotion concepts and entirely concrete action concepts would share the same systems, modelling the abstract/concrete difference by different brain systems appears as not fruitful.

A further possible point of criticism could be that we model concepts as static patterns only, whereas situational information associated with the experience of a scene over time is important. However, we do not believe that this is a valid criticism as the ‘instance patterns’ can each be seen as representations of situated information about an object, action or event. Going one step further, each individual instance could also be modelled as a range of similar but slightly different neural activation patterns, which could render the simulations one further step more realistic. The model mechanistically shows and explains how category representations can develop on the basis of variable situated experiences of objects, actions and events that to a degree share features.

### Conclusion

We here provide a neuroanatomically grounded computational model of the acquisition of concrete and abstract concepts through unsupervised Hebbian learning. The instances of concrete concepts were realized by overlapping sets of semantic features, whereas abstract concepts were realized by feature sets without common overlap, i.e. family resemblance. Robust neuronal conceptual representations emerged only in the case of concrete concepts. These circuits rely on large numbers of neurons in the neural network's central connector hub areas, which respond to shared semantic features of the conceptual category. For abstract concepts, more volatile representations emerged, consisting predominantly of unique, or idiosyncratic, feature neurons. Our findings also motivate novel hypotheses to be tested in future simulation and/or neuroimaging studies, in particular concerning a possible influence of verbal labels on conceptual learning.

## Data Availability

The datasets analyzed during the current study are available at https://osf.io/yvnsg/. An interactive version of Figure 3 is available at https://osf.io/cmhx6/.

## Appendix

### Structure and function of the spiking neuron model (adapted from Tomasello et al., 2018 and Garagnani et al., 2017)

Each of the 12 model areas consists of two layers of artificial neuron-like elements (“cells”), 625 excitatory and 625 inhibitory (*e*- and *i*-cells), thus resulting in 15,000 cells in total (see Fig. 1C). Each *e*-cell models a single representative pyramidal spiking neuron situated in a local patch of the cortex and the underlying *i*-cell represents the cluster of inhibitory interneurons located within the same cortical column (Eggert & van Hemmen, 2000; Wilson & Cowan, 1972). The state of each cell *x* at time *t* is uniquely defined by its membrane potential *V*(*x,t*), specified by the following equation:

1 $$\tau \cdot \frac{dV(x,t)}{dt}=-V(x,t)+{k}_{1}({V}_{In}(x,t)+{k}_{2}\eta (x,t))$$

where *V*_*In*_ (*x,t*) (defined by Eq. 2) is the net input acting upon cell *x* at time *t* (sum of all inhibitory and excitatory postsynaptic potentials—I/EPSPs; inhibitory synapses are given a negative sign), *τ* is the membrane’s time constant, *k*_1_, *k*_2_ are scaling values (see Table 1 for the specific parameter values used here) and *η*(*·*,*t*) is a white noise process with uniform distribution over [− 0.5,0.5].

**Table 1:** Parameter values used for the simulations

Equation (1)	Time constant (excitatory cells) Time constant (inhibitory cells) Total input rescaling factor Noise amplitude Global inhibition strength	*τ* = 2.5 (time steps) *τ* = 5 (time steps) *k*_1_ = 0.01 *k*_2_ = 7√(24/∆t) *k*_*G*_ = 0.80
Equation (3)	Spiking threshold	Thresh = 0.18
	Adaptation strength	*α* = 8.0
Equation (4)	Adaption time constant	*τ*_*ADAPT*_ = 10 (time steps)
Equation (5)	Rate-estimate time constant	*τ*_*Favg*_ = 30 (time steps, training) *τ*_*Favg*_ = 5 (time steps, testing)
Equation (6)	Global inhibition time constant	*τ*_*GLOB*_ = 12 (time steps)
Equation (7)	Postsynaptic potential thresholds for	*θ*_+_ = 0.15 (LTP) *θ*_−_ = 0.14 (LTD)
	Presynaptic output activity required for any synaptic change:	*θ*_*pre*_ = 0.05
	Learning rate	Δ*w* = 0.0008

Equation numbers refer to the equations in the appendix, where further details about the mathematical implementation of the model are described

**Table 2:** Connectivity structure of the modelled cortical areas

Modelled areas	References
Between-area connectivity (black arrows)	
Peri-sylvian system	
A1, AB, PB	Kaas and Hackett (2000), Pandya (1995), Rauschecker and Tian (2000)
PF_i_, PM_i_, M1_i_	Pandya and Yeterian (1985), Young et al., (1995)
Extra-sylvian system	
V1, TO, AT	Bressler et. al. (1993), Distler et. al. (1993)
PF_L_, PM_L_, M1_L_	Arikuni et. al. (1988), Dum and Strick (2002, 2005), Lu et. al. (1994), Pandya and Yeterian (1985), Rizzolatti and Luppino (2001)
Between system	
AT, PB	Gierhan (2013)
PF_i_, PF_L_	Yeterian et. al. (2012)
Long distance cortico-cortical connections (purple arrows)	
Peri-sylvian system	
PF_i_, PB	Catani et. al. (2005), Makris and Pandya (2009), Meyer et. al. (1999), Parker et. al. (2005), Paus et. al. (2001), Rilling et. al. (2008), Romanski et. al. (1999a, 1999b)
Extra-sylvian system	
AT, PF_L_	Bauer and Jones (1976), Chafee and Goldman-Rakic (2000), Eacott and Gaffan (1992), Fuster et. al. (1985), Parker (1998), Ungerleider et. al. (1989), Webster et. al. (1994)
Between system	
PB, PF_L_	Pandya and Barnes (1987); Romanski et. al. (1999a, 1999b), Romanski et. al. (1999a, 1999b)
AT, PF_i_	Pandya and Barnes (1987), Petrides and Pandya (2009), Rilling (2014), Romanski (2007), Ungerleider et. al. (1989), Webster et. al. (1994)
High-order “jumping” links (blue arrows)	
Peri-sylvian system (Rilling & Van Den Heuvel, 2018; Rilling et al., 2008, 2012; Thiebaut de Schotten et al., 2012)	
A1, PB	Pandya and Yeterian (1985), Young et. al. (1994)
PB, PM_i_	Rilling et. al. (2008), Saur et. al. (2008)
AB, PF_i_	Kaas and Hackett (2000), Petrides and Pandya (2009), Rauschecker and Scott (2009), Romanski et. al. (1999a, 1999b)
PFi, M1_i_	Deacon (1992), Guye et. al. (2003), Young et. al. (1995)
Extra-sylvian system (see also (Thiebaut de Schotten et al., 2012)	
V1, AT	Catani et al., (2003), Wakana et. al. (2004)
AT, PM_L_	Bauer and Fuster (1978), Chafee and Goldman-Rakic (2000), Fuster et. al. (1985), Pandya and Barnes (1987), Seltzer and Pandya (1989)
TO, PF_L_	Bauer and Jones (1976), Fuster and Jervey (1981), Fuster et. al. (1985), Makris and Pandya (2009), Seltzer and Pandya (1989)
PF_L_, M1_L_	Deacon (1992), Guye et. al. (2003), Young et. al. (1995)

Reprinted from Tomasello et al. (2018)

2 $${V}_{In}\left(\mathrm{x},\mathrm{t}\right)= -{k}_{G}{\upomega }_{\mathrm{G}}({A}_{x}, t) +\sum_{\forall \mathrm{y}}{\mathrm{w}}_{\mathrm{x},\mathrm{y}}\cdot \phi (y, t)$$

In Eq. (2), *y* varies over all cells in the network, *w*_*x*,*y*_ is the weight of the link from *y* to *x* and $\phi$ (*y*,*t*) is *y*’s current output (1 or 0), as defined below Eq. (3); *ω*_*G*_(*A*_*x*_,*t*) is the area-specific (or “global”) inhibition for area *A* where cell *x* is located (see explanation below and Eq. 6): this term is identical for all excitatory cells *x* in *A* and absent for inhibitory cells (*k*_*G*_ is a scaling constant). The weights of inhibitory synapses are assigned a negative sign. Note that noise is an inherent property of each model cell, intended to mimic the spontaneous activity (baseline firing) of real neurons. Therefore, noise was constantly present in all areas, in equal amounts (inhibitory cells have *k*_2_ = 0, i.e. the noise is generated by the excitatory cells). The output (or transformation function) *ϕ* of an excitatory cell *e* is defined as follows:

3 $$\phi \left( {e,t} \right)\left\{ {\begin{array}{*{20}c} 1 & {{\text{if}}\left( {V\left( {e,t} \right) - \alpha \omega \left( {e,t} \right)} \right) > {\text{thresh}}} \\ 0 & {{\text{otherwise}}\quad \quad \quad \quad \quad \quad \quad \quad } \\ \end{array} } \right.$$

Thus, an excitatory cell *e* spikes (= 1) whenever its membrane potential *V*(*e,t*) overcomes a fixed threshold *thresh* by the quantity *αω*(*e,t*) (where *α* is a constant and *ω* is defined below). Inhibitory cells are graded response neurons, for simplicity, as they intend to represent the average impact of a cluster of local interneurons; the output *ϕ*(*i,t*) of an inhibitory neuron *i* is 0 if *V*(*i,t*) < 0 and *V*(*i,t*) otherwise.

To simulate neuronal adaptation (Kandel et al., 2000), the function *ω*(*·,t*) is defined so as to track the cell’s most recent firing-rate activity. More precisely, the amount of adaptation (*e,t*) of cell *e* at time *t* is defined by:

4 $${\tau }_{ADAPT}\cdot \frac{d\omega \left(e,t\right)}{dt}=-\omega \left(e,t\right)+\phi (e,t)$$

where ${\tau }_{ADAPT}$ is the “adaptation” time constant. The solution (*e,t*) of Eq. (4) is the low-pass-filtered output *ϕ* of cell *e*, which provides an estimate of the cell’s most recent firing-rate history. A cell’s average firing activity is also used to specify the network’s Hebbian plasticity rule (see Eq. (7) below); in this context, the (estimated) instantaneous mean firing rate *ω*_*E*_(*e*,*t*) of an excitatory neuron *e* is defined as:

5 $${\tau }_{Favg}\cdot \frac{{d\omega }_{E}\left(e,t\right)}{dt}=-{\omega }_{E}\left(e,t\right)+\phi (e,t)$$

To regulate and control activity in the network, local and area-specific inhibition is implemented (Bibbig et al., 1995; Palm, 1982; Wennekers et al., 2006), realizing, respectively, local and global competition mechanisms (Duncan, 1996, 2006). More precisely, the input *V*_*In*_(*e,t*) (defined in Eq. 2) to each excitatory cell of the same area includes an area-specific (“global”) inhibition term *k*_*G*_*ω*_*G*_(*e*,*t*) (with *k*_*G*_ a constant and *ω*_*G*_(*e*,*t*) defined below) subtracted from the total I/EPSPs postsynaptic potentials *V*_*In*_ in input to the cell; this regulatory mechanism ensures that area (and network) activity is maintained within physiological levels (Braitenberg & Schüz, 1998):

6 $${\tau }_{GLOB}\cdot \frac{{d\omega }_{G}(e,t)}{dt}=-{\omega }_{G}(e,t)+{\sum }_{e\in area}\phi (e,t)$$

Excitatory links within and between (possibly non-adjacent) model areas are established at random and limited to a local (topographic) neighbourhood; weights are initialized at random, in the range [0, 0.1]. The probability of a synapse to be created between any two cells falls off with their distance (Braitenberg & Schüz, 1998) according to a Gaussian function clipped to 0 outside the chosen neighbourhood (a square of size *n* = 19 for excitatory and *n* = 5 for inhibitory cell projections). This produces sparse, patchy and topographic connectivity, as typically found in the mammalian cortex (Amir et al., 1993; Braitenberg & Schüz, 1998; Douglas & Martin, 2004; Kaas, 1997).

The Hebbian learning mechanism implemented simulates well-documented synaptic plasticity phenomena of long-term potentiation (LTP) and depression (LTD), as implemented by Artola, Bröcher and Singer (Artola & Singer, 1993; Artola et al., 1990). This rule provides a realistic approximation of known experience-dependent neuronal plasticity and learning (Finnie & Nader, 2012; Malenka & Bear, 2004; Musso et al., 1999; Rioult-Pedotti et al., 2000) and includes both (homo- and hetero-synaptic or associative) LTP, as well as homo- and hetero-synaptic LTD. In the model, we discretized the continuous range of possible synaptic efficacy changes into two possible levels, + Δ and − Δ (with Δ << 1 and fixed). Following Artola et al., we defined as “active” any (axonal) projection of excitatory cell *e* such that the estimated firing rate *ω*_*E*_(*e*,*t*) of cell *e* at time *t* (see Eq. (5)) is above *θ*_*pre*_, where *θ*_*pre*_ ∈]0,1] is an arbitrary threshold representing the minimum level of presynaptic activity required for LTP (or homosynaptic LTD) to occur. Thus, given a pre-synaptic cell *i* making contact on to a post-synaptic cell *j*, the change Δ*w*(*i,j*) inefficacy of the (excitatory-to-excitatory) link from *i* to *j* is calculated as follows:

7 $$\Delta w\left( {i,j} \right) = \left\{ {\begin{array}{*{20}c} { + \Delta } & {{\text{if }}\omega_{E} \left( {i,t} \right) \ge \theta_{pre} {\text{ and }}V\left( {j,t} \right) \ge \theta_{ + } \quad (LTP)\quad \quad \quad \quad \quad \quad \quad \quad } \\ { - \Delta } & {{\text{if }}\omega_{E} \left( {i,t} \right) \ge \theta_{pre} {\text{ and }}\theta_{ - } \le V\left( {j,t} \right) \ge \theta_{ + } \quad ({\text{homosynaptic }}LTD)} \\ { - \Delta } & {{\text{if }}\omega_{E} \left( {i,t} \right) < \theta_{pre} {\text{ and }}V\left( {j,t} \right) \ge \theta_{ + } \quad ({\text{heterosynaptic }}LTD)\quad \quad } \\ 0 & {{\text{otherwise}}\quad \quad \quad \quad \quad \quad \quad \quad \quad \quad \quad \quad \quad \quad \quad \quad \quad \quad \quad \quad \quad } \\ \end{array} } \right.$$

The values in Table 1 describe the parameters used during word learning simulation in the network, which were chosen on the basis of the previous simulations (e.g. Garagnani & Pulvermüller, 2011; Garagnani et al., 2007, 2009; Schomers et al., 2017; Tomasello et al., 2017).

## Footnotes

1 By ‘mind’, we mean cognitive and affective-emotional states and processes of a being, including those of conceptual thought, inference, prediction, memory, language, and attention, also including gestalt perception, coordinated motor movement, emotion experience and affect.

2 This section has been adapted from Tomasello et. al. (2019), as we used the same model architecture and connectivity features here.

3 This section has been adapted from Tomasello et al. (2019), as we used the same model architecture and connectivity features here.
